# Supplementary material for: Prevalence of Sleep Disturbance and Its Risk Factors in Patients Who Undergo Surgical Treatment for Degenerative Spinal Disease: A Nationwide Study of 106,837 Patients
Source: J Clin Med. 2022 Oct 8;11(19):5932. doi: 10.3390/jcm11195932 (PMC9572325; doi:10.3390/jcm11195932)
Supplement: Supplementary file 1 [file jcm-11-05932-s001.zip › jcm-1908602-supplementary.pdf]

Supplementary Table S1. Types of the used sleep medication

Category	Types of sleep medication	ATC code	HIRA general names	HIRA code
Benzodiazepines	triazolam	N05CD05	triazolam 0.125mg	243501ATB
		N05CD05	triazolam 0.25mg	243502ATB
	clonazepam	N03AE01	clonazepam 0.5mg	136401ATB
	flunitrazepam	N05CD03	flunitrazepam 1mg	160601ATB
	flurazepam	N05CD01	flurazepam hydrochloride 15mg	161801ATB
Benzodiazepine-related drug	zolpidem	N05CF02	zolpidem tartrate 10mg	250501ATB
		N05CF02	zolpidem tartrate 5mg	250502ATB
		N05CF02	zolpidem tartrate 6.25mg	250503ATR
		N05CF02	zolpidem tartrate 12.5mg	250504ATR
	eszopiclone	N05CF04	eszopiclone 1mg	680401ATB
		N05CF04	eszopiclone 2mg	680402ATB
		N05CF04	eszopiclone 3mg	680403ATB
TCA	Doxepin	D04AX01	doxepin hydrochloride (as doxepin 3mg)	149203ATB
		D04AX01	doxepin hydrochloride (as doxepin 6mg)	149204ATB

Supplementary Table S2. ICD-10 codes for comorbidities including Charlson comorbidities index items and scores

Type of comorbidities	Category	ICD-10 codes	Scores
Included in Charlson comorbidity index	Myocardial infarction	I21, I22, I25.2	1
	Congestive heart failure	I09.9, I11.0, I13.0, I13.2, I25.5, I42.0, I42.5-I42.9, I43, I50, P29.0	1
	Peripheral vascular disease	I70, I71, I73.1, I73.8, I73.9, I77.1, I79.0, I79.2, K55.1, K55.8, K55.9, Z95.8, Z95.9	1
	Cerebrovascular disease	G45, G46, I60-I69, H34.0	1
	Dementia	F00-F03, G30, F05.1, G31.1	1
	Chronic pulmonary disease	I27.8, I27.9, J40-J47, J60-J67, J68.4, J70.1, J70.3	1
	Rheumatologic disease	M05, M06, M31.5, M32-M34, M35.1, M35.3, M36.0	1
	Peptic ulcer	K25-K28	1
	Hemiplegia or paraplegia	G04.1, G11.4, G80.1, G80.2, G81, G82, G83.0, G83.1, G83.2, G83.3, G83.4, G83.9	2
	Diabetes without complication	E10.0, E10.1, E10.6, E10.8	1
	Diabetes with complication	E10.9, E11.0, E11.1, E11.6, E11.8, E11.9, E12.0, E12.1, E12.6, E12.8, E12.9, E13.0 E13.1, E13.6, E13.8, E13.9, E14.0, E14.1, E14.6, E14.8, E14.9	2
	Mild liver disease	B18, K70.0-K70.3, K70.9, K71.3-K71.5, K71.7, K73, K74, K76.0, K76.2-K76.4, K76.8, K76.9, Z94.4	1
	Moderate to severe liver disease	I85.0, I85.9, I86.4, I98.2, K70.4, K71.1, K72.1, K72.9, K76.5-K76.7	3
	Moderate to severe renal disease	I12.0, I13.1, N03.2-N03.7, N05.2-N05.7, N18, N19, N25.0, Z49.0-Z49.2, Z94.0, Z99.2	2
	Any malignancy (including leukemia or lymphoma)	C00-C26, C30-C34, C37-C41, C43, C45-C58, C60-C76 C81-C85, C88, C90-C97	2
	Metastatic solid tumor	C77-C80	6
	Acquired immunodeficiency syndrome	B20-B22, B24	6
Additional neuropsychiatric disorders associated with sleep disturbance	Parkinson disease	G20	-

	Migraine	G43	-
	Tension type headache	G44.2	-
	Other-type headache	G44.0, G44.1, G44.3, G44.4, G44.8	-
Others	Osteoporosis	M80-M82	-
	End stage renal disease	E10.22, E11.22, E12.22, E13.32, E14.22, N18.5, Z99.2 (V001, V003)	-

Supplementary Table S3. ATC and HIRA codes for the used antidepressant

Types of sleep medication	ATC code	HIRA general names	HIRA code
agomelatine	N06AX22	agomelatine 25mg	613101ATB
amitriptyline	N06AA09	amitriptyline 10mg	107501ATB
amitriptyline	N06AA09	amitriptyline 25mg	107502ATB
amitriptyline	N06AA09	amitriptyline 5mg	107504ATB
amoxapine	N06AA17	amoxapine 50mg	108002ATB
bupropion	N06AX12	bupropion 0.1g	428101ATB
bupropion	N06AX12	bupropion 0.15g	428102ATR
bupropion	N06AX12	bupropion 0.3g	428103ATR
citalopram	N06AB04	citalopram 24.99mg	428301ATB
clomipramine	N06AA04	clomipramine 10mg	136301ACH
clomipramine	N06AA04	clomipramine 25mg	136302ACH
desvenlafaxine	N06AX23	desvenlafaxine 75.87mg	626401ATR
desvenlafaxine	N06AX23	desvenlafaxine 0.15177g	626402ATR
doxepin	N06AA12	doxepin 3.39mg	149203ATB
doxepin	N06AA12	doxepin 6.78mg	149204ATB
duloxetine	N06AX21	duloxetine 0.1765g	495501ACE
duloxetine	N06AX21	duloxetine 33.65mg	495501ATE
duloxetine	N06AX21	duloxetine 67.3mg	495502ACE
duloxetine	N06AX21	duloxetine 67.3mg	495502ATE
escitalopram	N06AB10	escitalopram 5mg	474801ATB
escitalopram	N06AB10	escitalopram 12.77mg	474802ATB
escitalopram	N06AB10	escitalopram 20mg	474803ATB
escitalopram	N06AB10	escitalopram 15mg	474804ATB
fluoxetine	N06AB03	fluoxetine 11.2mg	161501ACH
fluoxetine	N06AB03	fluoxetine 11.2mg	161501ATB
fluoxetine	N06AB03	fluoxetine 22.4mg	161502ACH
fluoxetine	N06AB03	fluoxetine 22.4mg	161502ATB
fluoxetine	N06AB03	fluoxetine 22.37mg	161502ATD
fluvoxamine	N06AB08	fluvoxamine 50mg	162501ATB
fluvoxamine	N06AB08	fluvoxamine 0.1g	162502ATB
Hyperici herba	N06AX25	Hyperici herba 0.25g	149901ATB
Hyperici herba	N06AX25	Hyperici herba 0.3g	172601ATB
imipramine	N06AA02	imipramine 25mg	173701ATB
milnacipran	N06AX17	milnacipran 25mg	355801ACH
milnacipran	N06AX17	milnacipran 50mg	355802ACH
milnacipran	N06AX17	milnacipran 12.5mg	355803ACH
mirtazapine	N06AX11	mirtazapine 15mg	196201ATB
mirtazapine	N06AX11	mirtazapine 15mg	196201ATD
mirtazapine	N06AX11	mirtazapine 30mg	196202ATB
mirtazapine	N06AX11	mirtazapine 30mg	196202ATD
mirtazapine	N06AX11	mirtazapine 7.5mg	196204ATB
mirtazapine	N06AX11	mirtazapine 7.5mg	196204ATD
moclobemide	N06AG02	moclobemide 0.15g	196701ATB
nortriptyline	N06AA10	nortriptyline 11.4mg	203401ATB
nortriptyline	N06AA10	nortriptyline 28.5mg	203402ATB
paroxetine	N06AB05	paroxetine 11.4mg	209301ATB
paroxetine	N06AB05	paroxetine 22.8mg	209302ATB
paroxetine	N06AB05	paroxetine 14.25mg	209304ATR
paroxetine	N06AB05	paroxetine 28.5mg	209305ATR

sertraline	N06AB06	sertraline 55.95mg	227001ATB
sertraline	N06AB06	sertraline 55.95mg	227001ATB
sertraline	N06AB06	sertraline 0.1119g	227002ATB
sertraline	N06AB06	sertraline 27.98mg	227003ATB
tianeptine	N06AX14	tianeptine 12.5mg	229601ATB
trazodone	N06AX05	trazodone 25mg	242901ACH
trazodone	N06AX05	trazodone 25mg	242901ATB
trazodone	N06AX05	trazodone 50mg	242902ATB
trazodone	N06AX05	trazodone 75mg	242903ATR
venlafaxine	N06AX16	venlafaxine 84.84mg	247502ACR
venlafaxine	N06AX16	venlafaxine 42.42mg	247504ACR
vortioxetine	N06AX26	vortioxetine 6.355mg	628501ATB
vortioxetine	N06AX26	vortioxetine 12.71mg	628502ATB
vortioxetine	N06AX26	vortioxetine 25.42mg	628504ATB

Supplementary Table S4. HIRA codes for the x-rays of the extremities

Extremities	HIRA codes for the x-rays of the extremities
Shoulder	G3301, G3302, G3303, G3304, G3305, G3311, G3312, G3313, G3314, G3315, G3321, G3322, G3323, G3324, G3325
Elbow	G6201, G6202, G6203, G6204, G6205, G6211, G6212, G6213, G6214, G6215, G6221, G6222, G6223, G6224, G6225
Wrist	G6401, G6402, G6403, G6404, G6405, G6411, G6412, G6413, G6414, G6415, G6421, G6422, G6423, G6424, G6425
Hip	G5201, G5202, G5203, G5204, G5205, G5211, G5212, G5213, G5214, G5215, G5221, G5222, G5223, G5224, G5225
Knee	G7201, G7202, G7203, G7204, G7205, G7211, G7212, G7213, G7214, G7215, G7221, G7222, G7223, G7224, G7225
Ankle	G7401, G7402, G7403, G7404, G7405, G7411, G7412, G7413, G7414, G7415, G7421, G7422, G7423, G7424, G7425

Supplementary Table S5. Risk factors for sleep disorder (main analysis): all the results from statistical analysis

Variables	Categories	Univariable		Model 2 (Fully adjusted)		Model 3 (bootstrap validation after fully adjusted)	
		Odds ratio (95% confidence interval)	p-value	Adjusted odds ratio (95% confidence interval)	p-value	Adjusted odds ratio (95% confidence interval)	Relative bias (%)
Age	50-69 vs 20-49 years	1.85 [1.66–2.06]	<0.001	1.40 [1.25–1.57]	<0.001	1.41 [1.29–1.56]	2.21
	70-79 vs 20-49 years	2.87 [2.57–3.21]	<0.001	1.80 [1.60–2.03]	<0.001	1.81 [1.64–2.04]	0.89
	80+ vs 20-49 years	3.50 [3.05–4.02]	<0.001	2.22 [1.92–2.58]	<0.001	2.23 [1.96–2.55]	0.80
Sex	Female vs male	1.45 [1.38–1.53]	<0.001	1.14 [1.07–1.21]	<0.001	1.13 [1.08–1.20]	-4.45
Region	Urban vs rural	1.13 [1.05–1.22]	0.001	1.18 [1.09–1.27]	<0.001	1.17 [1.11–1.24]	-2.97
Hospital	Tertiary vs others	1.25 [1.16–1.34]	<0.001	1.08 [1.00–1.16]	0.047	1.08 [1.00–1.15]	-3.33
	General vs others	1.20 [1.13–1.29]	<0.001	1.06 [0.99–1.13]	0.121	1.06 [1.00–1.13]	-3.55
Charlson comorbidity index score	3-5 vs 0-2	1.89 [1.77–2.02]	<0.001	1.07 [0.96–1.20]	0.232	1.07 [0.97–1.18]	4.64
	≥6 vs 0-2	2.42 [1.97–2.99]	<0.001	0.99 [0.74–1.34]	0.969	0.99 [0.76–1.29]	33.33
Comorbidities	Myocardial infarction	1.40 [1.10–1.78]	0.007	1.20 [0.93–1.54]	0.159	1.17 [0.91–1.48]	-14.38
	Congestive heart failure	1.62 [1.43–1.83]	<0.001	1.10 [0.97–1.26]	0.144	1.11 [0.99–1.24]	5.86
	Peripheral vascular disease	1.61 [1.50–1.73]	<0.001	1.22 [1.13–1.32]	<0.001	1.22 [1.13–1.31]	0.68
	Chronic pulmonary disease	1.66 [1.57–1.76]	<0.001	1.31 [1.23–1.40]	<0.001	1.30 [1.23–1.38]	-1.42
	Rheumatologic disease	1.38 [1.22–1.56]	<0.001	1.04 [0.91–1.18]	0.589	1.04 [0.94–1.13]	-2.09
	Peptic ulcer disease	1.60 [1.50–1.70]	<0.001	1.26 [1.17–1.35]	<0.001	1.26 [1.19–1.34]	-0.91
	Liver disease						
	Mild	1.38 [1.26–1.52]	<0.001	1.27 [1.14–1.41]	<0.001	1.26 [1.17–1.37]	-1.68
	Moderate to severe	1.60 [0.74–3.46]	0.234				
	Diabetes						
	Uncomplicated	1.26 [1.18–1.34]	<0.001	1.02 [0.95–1.10]	0.567	1.02 [0.96–1.09]	8.57

	Complicated	1.21 [1.09–1.33]	<0.001	0.93 [0.82–1.06]	0.299	0.92 [0.78–1.08]	10.79
	Hemiplegia or paraplegia	1.08 [0.81–1.44]	0.592				
	Renal disease	1.67 [1.43–1.95]	<0.001	1.20 [0.98–1.46]	0.075	1.20 [1.00–1.41]	-1.82
	End stage renal disease	1.99 [1.43–2.77]	<0.001	1.46 [1.00–2.14]	0.053	1.44 [0.95–2.02]	-3.84
	Osteoporosis	1.54 [1.44–1.65]	<0.001	1.04 [0.97–1.12]	0.316	1.04 [0.98–1.11]	-8.72
Comorbidities associated neuropsychiatric disorders	Depressive disorder	3.52 [3.34–3.72]	<0.001	2.86 [2.70–3.02]	<0.001	2.86 [2.72–3.00]	0.03
	Cerebrovascular disease	1.70 [1.58–1.84]	<0.001	1.12 [1.10–1.20]	0.040	1.10 [1.02–1.19]	-16.00
	Dementia	2.41 [2.04–2.83]	<0.001	1.49 [1.26–1.78]	<0.001	1.50 [1.32–1.71]	0.96
	Parkinson disease	2.25 [1.83–2.78]	<0.001	1.51 [1.22–1.88]	<0.001	1.50 [1.21–1.83]	-1.63
	Migraine	2.43 [2.18–2.71]	<0.001	1.61 [1.44–1.82]	<0.001	1.62 [1.45–1.79]	1.76
	Tension type headache	2.30 [2.05–2.58]	<0.001	1.20 [0.96–1.50]	0.105	1.21[1.01–1.44]	3.33
	Other-type headache	2.21 [2.00–2.44]	<0.001	1.25 [1.03–1.52]	0.023	1.24 [1.06–1.44]	-2.75
Surgical regions	Cervical vs lumbar	0.77 [0.72–0.83]	<0.001	1.00 [0.92–1.08]	0.929	0.99 [0.92–1.07]	-23.39
	Thoracic vs lumbar	1.23 [0.97–1.57]	0.089	1.13 [0.88–1.45]	0.326	1.13 [0.89–1.41]	1.79
Concurrent osteoarthritis	Shoulder	1.55 [1.43–1.69]	<0.001	1.15 [1.06–1.26]	0.002	1.15 [1.08–1.24]	1.17
	Elbow	1.14 [0.96–1.36]	0.126				
	Wrist	1.53 [1.32–1.79]	<0.001	1.13 [0.96–1.32]	0.145	1.11 [0.96–1.28]	-12.29
	Hip	1.47 [1.34–1.61]	<0.001	1.05 [0.95–1.15]	0.352	1.04 [0.97–1.12]	-12.28
	Knee	1.59 [1.50–1.68]	<0.001	1.11 [1.04–1.18]	0.002	1.11 [1.05–1.17]	-1.40
	Ankle	1.79 [1.60–2.00]	<0.001	1.32 [1.17–1.48]	<0.001	1.32 [1.18–1.46]	0.67

Relative bias was estimated as the difference between the mean bootstrapped regression coefficient estimates (model 3) and the mean parameter estimates of multivariable model (model 2) divided by the mean parameter estimates of multivariable model (model 2).

Supplementary Table S6. Risk factors for over 8-week sleep medication during the preoperative 90 days (sensitivity analysis): all the results from statistical analysis

Variables	Categories	Univariable		Model 2 (Fully adjusted)		Model 3 (bootstrap validation after fully adjusted)	
		Odds ratio (95% confidence interval)	p-value	Adjusted odds ratio (95% confidence interval)	p-value	Adjusted odds ratio (95% confidence interval)	Relative bias (%)
Age	50-69 vs 20-49 years	1.97 [1.77–2.21]	<0.001	1.32 [1.17–1.49]	<0.001	1.31 [1.19–1.44]	-2.60
	70-79 vs 20-49 years	3.04 [2.71–3.41]	<0.001	1.54 [1.36–1.75]	<0.001	1.53 [1.37–1.71]	-0.78
	80+ vs 20-49 years	3.80 [3.31–4.37]	<0.001	1.95 [1.68–2.27]	<0.001	1.94 [1.71–2.22]	-0.69
Sex	Female vs male	1.57 [1.49–1.66]	<0.001	1.20 [1.13–1.27]	<0.001	1.19 [1.14–1.26]	-3.96
Region	Urban vs rural	1.04 [0.97–1.11]	0.328				
Hospital	Tertiary vs others	1.38 [1.29–1.48]	<0.001	1.17 [1.08–1.15]	<0.001	1.16 [1.09–1.24]	-4.46
	General vs others	1.63 [1.53–1.74]	<0.001	1.38 [1.29–1.47]	<0.001	1.38 [1.30–1.46]	-0.34
Charlson comorbidity index score	3-5 vs 0-2	2.16 [2.02–2.30]	<0.001	1.05 [0.94–1.19]	0.391	1.05 [0.94–1.18]	1.04
	≥6 vs 0-2	2.42 [1.97–2.99]	<0.001	0.99 [0.74–1.33]	0.947	0.98 [0.72–1.26]	112.93
Comorbidities	Myocardial infarction	1.41 [1.11–1.80]	0.005	1.15 [0.89–1.48]	0.296	1.15 [0.92–1.43]	-1.48
	Congestive heart failure	1.80 [1.60–2.03]	<0.001	1.16 [1.02–1.33]	0.023	1.17 [1.06–1.32]	6.99
	Peripheral vascular disease	1.53 [1.42–1.65]	<0.001	1.09 [1.00–1.18]	0.040	1.09 [1.01–1.18]	-0.67
	Chronic pulmonary disease	1.60 [1.51–1.69]	<0.001	1.21 [1.13–1.29]	<0.001	1.20 [1.13–1.27]	-4.26
	Rheumatologic disease	1.40 [1.24–1.58]	<0.001	1.00 [0.88–1.14]	0.975	1.00 [0.91–1.13]	-66.72
	Peptic ulcer disease	1.58 [1.48–1.68]	<0.001	1.20 [1.11–1.29]	<0.001	1.20 [1.13–1.29]	-0.34
	Liver disease						
	Mild	1.42 [1.29–1.56]	<0.001	1.27 [1.14–1.40]	<0.001	1.27 [1.17–1.39]	-0.13
	Moderate to severe	2.10 [1.05–4.19]	0.036	1.74 [0.83–3.69]	0.146	1.70 [0.84–3.28]	-4.26
	Diabetes						
	Uncomplicated	1.46 [1.38–1.55]	<0.001	1.12 [1.04–1.20]	0.002	1.11 [1.05–1.19]	-4.17

	Complicated	1.62 [1.48–1.77]	<0.001	1.05 [0.92–1.20]	0.502	1.05 [0.93–1.16]	-8.38
	Hemiplegia or paraplegia	1.55 [1.22–1.99]	<0.001	1.06 [0.81–1.39]	0.684	1.05 [0.81–1.36]	-21.57
	Renal disease	2.01 [1.74–2.32]	<0.001	1.23 [1.01–1.49]	0.042	1.22 [1.01–1.48]	-5.03
	End stage renal disease	3.07 [2.31–4.07]	<0.001	1.97 [1.39–2.79]	<0.001	1.96 [1.45–2.71]	-0.76
	Osteoporosis	1.54 [1.44–1.65]	<0.001	0.98 [0.91–1.05]	0.545	0.98 [0.92–1.04]	-23.28
Comorbidities associated neuropsychiatric disorders	Depressive disorder	7.05 [6.67–7.45]	<0.001	5.84 [5.51–6.18]	<0.001	5.84 [5.57–6.16]	0.01
	Cerebrovascular disease	2.15 [2.00–2.31]	<0.001	1.28 [1.18–1.39]	<0.001	1.28 [1.19–1.38]	0.54
	Dementia	2.28 [1.93–2.69]	<0.001	1.33 [1.11–1.59]	0.002	1.32 [1.13–1.55]	-1.68
	Parkinson disease	4.41 [3.73–5.21]	<0.001	2.80 [2.34–3.36]	<0.001	2.83 [2.46–3.32]	1.00
	Migraine	2.21 [1.98–2.48]	<0.001	1.30 [1.15–1.47]	<0.001	1.31 [1.18–1.44]	1.88
	Tension type headache	2.18 [1.94–2.45]	<0.001	1.23 [0.98–1.55]	0.080	1.23 [1.00–1.51]	-0.97
	Other-type headache	2.09 [1.89–2.31]	<0.001	1.03 [0.84–1.25]	0.801	1.02 [0.86–1.19]	-26.59
Surgical regions	Cervical vs lumbar	0.74 [0.69–0.80]	<0.001	0.94 [0.86–1.02]	0.133	0.94 [0.87–1.01]	5.05
	Thoracic vs lumbar	1.07 [0.83–1.39]	0.584	0.91 [0.70–1.19]	0.506	0.91 [0.70–1.15]	2.96
Concurrent osteoarthritis	Shoulder	1.47 [1.35–1.60]	<0.001	1.04 [0.95–1.14]	0.430	1.03 [0.96–1.12]	-13.31
	Elbow	0.97 [0.80–1.16]	0.719				
	Wrist	1.61 [1.39–1.87]	<0.001	1.16 [0.99–1.36]	0.074	1.15 [1.01–1.32]	-5.40
	Hip	1.43 [1.30–1.57]	<0.001	0.95 [0.86–1.05]	0.302	0.96 [0.88–1.04]	-10.51
	Knee	1.68 [1.58–1.77]	<0.001	1.14 [1.06–1.21]	<0.001	1.13 [1.07–1.19]	-5.13
	Ankle	1.70 [1.52–1.90]	<0.001	1.19 [1.06–1.35]	0.004	1.20 [1.09–1.32]	5.14

Relative bias was estimated as the difference between the mean bootstrapped regression coefficient estimates (model 3) and the mean parameter estimates of multivariable model (model 2) divided by the mean parameter estimates of multivariable model (model 2).
